# Swimming as Treatment of Scapular Dyskinesis

**DOI:** 10.1155/2019/5607970

**Published:** 2019-01-20

**Authors:** Max Rogerio Freitas Ramos, Yonder Archanjo Ching San Junior, Leonardo Antunes Bellot de Souza

**Affiliations:** ^1^HUGG-UNIRIO, Brazil; ^2^UNIRIO, Brazil

## Abstract

Scapular dyskinesis is quite frequent and can lead to shoulder pain. The diagnosis is essentially clinical. The main cause is muscle imbalance, between the trapezius, rhomboids, and pectoralis minor. In these cases, rehabilitation is the best treatment. We present a case of a young male patient with dyskinesis due to axonal involvement of the long thoracic nerve and paresis of the anterior serratus muscle. After a swimming program to increase muscular strength and imbalance, he experienced pain reduction and functional recovery of the upper limb, with reduction of the winged scapula.

## 1. Introduction

The scapular girdle comprises a complex osteomuscular and articular system that aims to allow wide movement and functionality of the upper limb [1]. The scapula acts as a stabilizer and fixed point of muscular insertions that allows shoulder movement. It provides the transfer of strength from the limb to the trunk and the wide range of motion [1–3].

The scapulothoracic joint is responsible for a third of the shoulder movement [2, 3]. The relationship between the scapula and the thoracic wall through the action of stabilizing muscles allows a complex three-dimensional movement that results in the wide mobility of the upper limb [3].

Scapular dyskinesis is not a pathological condition but an alteration of the biomechanical and articular relationship [2–4]. Among other causes, muscular imbalance plays the main role [2]. The imbalance between the trapezius, serratus, levator scapulae, and pectoralis minor results in dyskinesis. Bone alterations, such as shortening of the clavicle or acromioclavicular incongruity, are frequent. Neurological causes such as long thoracic nerve paralysis or tumor constitute differential diagnoses [2–4]. Treatment, in most cases, is conservative [1–5]. The aim of the treatment is neuromuscular rehabilitation, with strengthening of the dorsal periscapular musculature and shortening of the pectoralis minor, thus improving harmonic movement of this joint.

## 2. Case Report

A 19-year-old male patient complained of shoulder pain with no trauma history. He reported that the pain started about 6 months before, with progressive worsening. Pain was present in elevation and abduction, especially above 90°. The pain caused him to interrupt his physical activities, although he did not notice worsening during bodybuilding.

**Figure 1:**
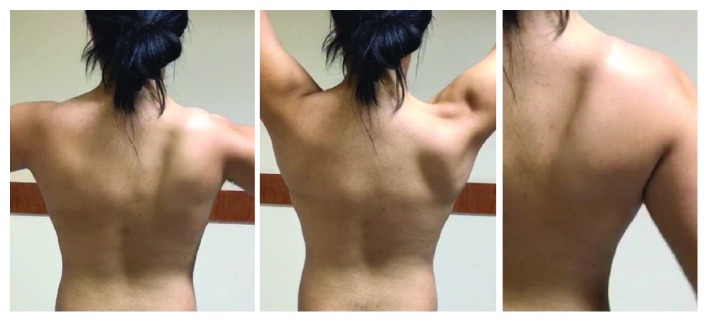
Scapular dyskinesis.

A winged scapula was identified in physical exam, with scapulothoracic grade III dyskinesia, according to Kibler et al. [3]. He presented infraspinatus atrophy, complete range of motion, preserved upper limb strength, and discrete paraesthesia at the region of the medial border of the right scapula, with no other signs. There were no clinical signs suggestive of rotator cuff injury or glenohumeral instability (Figure 1).

The imaging exams did not show significant changes. Shoulder MRI showed no rotator cuff lesion, labral lesions, cysts, or other soft tissue involvement. The cervical spine MRI did not show cervical discopathy or syringomyelia. Electroneuromyography with evoked potential of the scapular girdle evidenced diffuse axonal involvement of the long thoracic nerve, without other alterations.

The patient was then referred to the physiotherapy service, where he initiated a program of shoulder girdle rehabilitation focused on analgesia and passive mobilization. During ten weeks, he remained under the care of physiotherapists twice a week, but he did not notice an improvement in the pain. When he returned to the orthopedic clinic, he was informed about the possibility of surgical treatment.

The patient insisted on conservative treatment. We recommended strengthening of the shoulder girdle and swimming. During three months, he practiced swimming three times a week under the guidance of a physical education professional with experience in athlete training. In order to strengthen the periscapular muscles, he tried to practice the four classic styles of swimming, using floats in the lower limbs and increasing the demand on the upper limbs. Progressively, he noticed an improvement in his pain.

The swimming program consists of a 60-minute pool training three times a week, with increasing distances. The front crawl, breaststroke, and backstroke were alternated during training. The main set was a target mile, split in 200 m lengths with 1-minute rest (in a 25 m pool) and alternating strokes.

**Figure 2:**
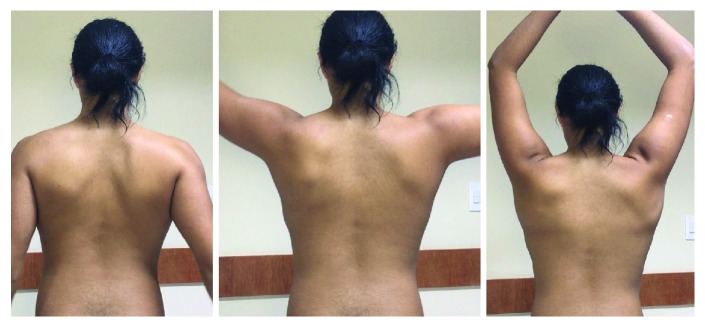
The result after the muscular equilibrium and swimming rehabilitation program coordinated by a physical rehabilitation specialist.

He returned after 90 days, free of pain. He presented dynamic stabilization of the scapula during elevation, and dyskinesia was no longer perceived. The force remained unchanged, but atrophy was no longer identified. The patient was satisfied with the progress made and was encouraged to stay in the muscle strengthening program in aquatic activities (Figure 2).

## 3. Discussion

The scapula is the point of origin or insertion of 17 muscles and acts as a fixed point of the stabilizing muscles, depressors, and rotators of the humeral head [2]. The rotation, elevation, and translation of the scapula relative to the trunk during the movement of the elevation allow a wide elevation through the alteration of the scapulothoracic angle; at the same time, the scapula acts as anchorage of the rotator cuff [1–5].

The shoulder girdle articulates with the chest wall through the sternoclavicular joint and the scapulothoracic joint. The clavicle provides the only bone connection of the scapula to the axial skeleton through the acromioclavicular and sternoclavicular joints. In the scapulothoracic joint, there is no bone congruence, and stability is achieved through muscular imbalance. The articulation of the scapula with the thoracic wall allows for complex three-dimensional movements and is responsible for one-third of the range of motion, with the glenohumeral joint accounting for two-thirds (120°) [4–6].

The complex movement of the scapulothoracic joint occurs throughout the entire arch of shoulder elevation. When the upper limb is erected above the head, the scapula undergoes superior rotation and translation, internal rotation initially, and subsequently external rotation and protrusion (posterior inclination), associated with elevation and retraction of the clavicle. This three-dimensional displacement on the cylindrical surface of the thorax allows stable elevation of the upper limb and force transmission [4, 7, 8].

Dyskinesis occurs when the movement of the scapula relative to the thorax does not occur in a harmonic way, leading to overload of the glenohumeral joint and the various muscle groups involved, causing pain and restriction of movement [7–9].

The diagnosis is essentially clinical, through the static and dynamic inspection of the scapula [3]. Dynamic tests, retraction test, and measurement of elevation and inclination angles provide objective parameters [3–7]. Imaging examinations are useful for the diagnosis of pseudoarthrosis, fractures, or arthrosis in the clavicle and scapula and of neoplastic affections. Electromyography is useful to evaluate the neural involvement, especially the long thoracic nerve and spinal accessory nerve [5].

The main cause of dyskinesia is muscle imbalance. In addition, thoracic kyphosis, scoliosis, pseudoarthrosis or malunion of clavicle fracture with shortening, acromioclavicular instability, acromioclavicular arthrosis, changes in the glenohumeral joint, cervical radiculopathy, paralysis of the long thoracic or spinal accessory nerves, stiffness, muscle contractions, shortening of the pectoralis minor or short head of the biceps, adhesive capsulitis, internal rotation deficiency, impingement syndrome, and rotator cuff disorders are the main etiologies [4, 7, 8].

Scapular dyskinesis is classified into four different patterns. Type I is characterized by the prominence of the inferomedial border of the scapula by the abnormal inclination on the horizontal axis. Type II consists of the prominence of the entire medial border due to the external rotation in the vertical plane. Type III presents as rotation of the superomedial border on a horizontal axis perpendicular to the plane of the scapula, resulting in superior migration of the scapula. Type IV consists of a mixed dyskinesis [3, 8, 9].

The treatment consists mainly of rehabilitation, focusing on muscular rebalancing, even in asymptomatic patients, thus preventing the onset of injuries. The primary goal is to restore the dynamic balance of the muscular forces acting on the scapula. In addition, the elongation of the posteroinferior capsule and the pectoralis minor muscle is essential to achieve imbalance [2, 5–9].

## 4. Conclusion

Scapulothoracic dyskinesis is a relatively common condition, although underdiagnosed and eventually resulting in clinical repercussions. Rehabilitation is the mainstay of treatment. Correct diagnosis and muscle rebalancing help relieve pain and complications and allow the return to sports and physical activities.
